# Studying Cat (*Felis catus*) Diabetes: Beware of the Acromegalic Imposter

**DOI:** 10.1371/journal.pone.0127794

**Published:** 2015-05-29

**Authors:** Stijn J. M. Niessen, Yaiza Forcada, Panagiotis Mantis, Christopher R. Lamb, Norelene Harrington, Rob Fowkes, Márta Korbonits, Ken Smith, David B. Church

**Affiliations:** 1 Department of Clinical Science and Services, Royal Veterinary College, University of London, London, United Kingdom; 2 The Diabetes Research Group, Institute of Cellular Medicine, University of Newcastle, Newcastle, Tyne and Wear, United Kingdom; 3 Department of Pathology and Pathogen Biology, Royal Veterinary College, University of London, London, United Kingdom; 4 Department of Comparative Biology, Royal Veterinary College, University of London, London, United Kingdom; 5 Department of Endocrinology, Barts & the Royal London School of Medicine & Dentistry, WHRI, Queen Mary University of London, London, United Kingdom; John Hopkins University School of Medicine, UNITED STATES

## Abstract

Naturally occurring diabetes mellitus (DM) is common in domestic cats (*Felis catus*). It has been proposed as a model for human Type 2 DM given many shared features. Small case studies demonstrate feline DM also occurs as a result of insulin resistance due to a somatotrophinoma. The current study estimates the prevalence of hypersomatotropism or acromegaly in the largest cohort of diabetic cats to date, evaluates clinical presentation and ease of recognition. Diabetic cats were screened for hypersomatotropism using serum total insulin-like growth factor-1 (IGF-1; radioimmunoassay), followed by further evaluation of a subset of cases with suggestive IGF-1 (>1000 ng/ml) through pituitary imaging and/ or histopathology. Clinicians indicated pre-test suspicion for hypersomatotropism. In total 1221 diabetic cats were screened; 319 (26.1%) demonstrated a serum IGF-1>1000 ng/ml (95% confidence interval: 23.6–28.6%). Of these cats a subset of 63 (20%) underwent pituitary imaging and 56/63 (89%) had a pituitary tumour on computed tomography; an additional three on magnetic resonance imaging and one on necropsy. These data suggest a positive predictive value of serum IGF-1 for hypersomatotropism of 95% (95% confidence interval: 90–100%), thus suggesting the overall hypersomatotropism prevalence among UK diabetic cats to be 24.8% (95% confidence interval: 21.2–28.6%). Only 24% of clinicians indicated a strong pre-test suspicion; most hypersomatotropism cats did not display typical phenotypical acromegaly signs. The current data suggest hypersomatotropism screening should be considered when studying diabetic cats and opportunities exist for comparative acromegaly research, especially in light of the many detected communalities with the human disease.

## Introduction

Spontaneous diabetes mellitus (DM) in the domestic cat (*Felis catus)* is currently estimated to affect approximately 1 in 200 household cats in the UK [1] and between 1 and one and a half in 200 household cats in Australia [2]. This number, in concordance with human DM, is rapidly rising. For example, in the United States estimates rose from less than a half per 200 in 1970 to approximately two and a half in 200 in 1999 [3]. In addition to therefore being an important endocrinopathy in veterinary medicine, feline DM has been increasingly studied in the field of comparative endocrinology as a spontaneous model for human type 2 DM based on a multitude of shared characteristics. Indeed, domestic cats share the same environment as their human counterparts and, akin to the situation in man, the diabetic cat seems to suffer from a combination of insulin resistance and beta-cell dysfunction [4–6]. Additionally, like in humans, diabetic cats generally are middle-aged at time of diagnosis and both obesity and inactivity pose significant risk factors for its development [1,4,5]. Furthermore, similarities exist in the genetic predisposition for human type 2 DM and feline DM. Polymorphisms in the melanocortin receptor 4 gene are associated with DM in both overweight cats and humans [7]. Finally, man and cat share histopathological features of Type 2 DM with deposition of pancreatic islet amyloid being demonstrable in both species [5,8–10].

However, although this feline type 2 DM is suspected to be the most common type of spontaneous DM in the cat, other types of spontaneous feline DM have also been described to occur, particularly DM as a consequence of endogenous overproduction of hormones with an insulin-antagonistic effect such as cortisol (hyperadrenocorticism or Cushing’s syndrome) and growth hormone (HS or acromegaly) [11]. Relatively small case series have described HS in cats and to date, only one small scale pilot screening study has been performed, which in fact hinted towards HS-induced DM to be unexpectedly common among diabetic cats, with 59 out of 184 diabetic cats (32%) showing evidence of the disease [12]. Data on pre-test clinical suspicion for HS were not included in this small study. Interestingly, all reported feline acromegalics have been diabetic, in contrast to the situation in man, where acromegaly induces DM only in a proportion of patients [11,13,14].

Confirmation of a high prevalence of this different type of DM among a larger cohort of diabetic cats would have a considerable impact on the use of feline DM in comparative endocrinology research, the overall understanding of feline DM and the approach of the veterinary profession to its management. This impact would be even greater, if the feline syndrome proved difficult to recognise phenotypically and therefore would be missed without routine laboratory screening. Finally, a high prevalence of feline acromegaly would mean attractive opportunities exist for comparative somatotrophinoma research.

In light of the initial pilot data [12], a complete study seemed warranted and, therefore, the current study aimed to quantify the true potential relative contribution of naturally occurring HS in the etiology of spontaneous feline diabetes mellitus through the screening of the largest cohort of diabetic pet cats ever reported. In order to further investigate the need for routine HS screening of diabetic cats, the study also aimed to assess pre-test suspicion among veterinary professionals, as well as to compare clinical features of diabetic acromegalic and diabetic non-acromegalic cats.

## Materials and Methods

### Recruitment of diabetic cats

Serum fructosamine concentration is a reflection of average level of glycaemia over the preceding two to three weeks in cats and is currently routinely used by veterinarians in the diagnosis of DM and the subsequent evaluation of glycaemic control after the start of insulin treatment. From October 2003 till April 2011 evaluation of serum fructosamine was offered free of charge for all diabetic cats attending any veterinary practice in the UK. This service was widely and continuously advertised to the veterinary profession around the UK through multiple channels of communication (websites, newsletters, regional and national conference presentations). Cats were defined as diabetic on the basis of documentation of clinical signs suggestive of diabetes mellitus and persistent elevation of fasted blood glucose concentrations. At the time of sample submission, attending veterinarians were asked to record clinical data about the patient, including age, breed, gender, current body weight, current administered insulin dose (considered part of standard treatment for feline DM) and whether they clinically suspected acromegaly/ hypersomatotropism. The latter suspicion was categorized into 3 options (1. Yes, strongly; 2. No; 3. Maybe). Excess serum was used to evaluate basal total IGF-1 concentration in all received diabetic cat samples.

### Diagnosis of hypersomatotropism

Diabetic cats were classified as suspected HS-induced DM on the basis of elevation of serum basal total IGF-1 concentration above 1000 ng/ml. At this cut-off serum IGF-1 was previously shown to have a positive predictive value of 95% for the presence of HS in the diabetic cat [11,12]. All veterinarians dealing with a cat with a serum IGF-1 concentration that exceeded 1000 ng/mL, were sent a standard letter explaining the cat could be suffering from hypersomatotropism and inviting the owner to attend a dedicated Veterinary Endocrinology Clinic run by board-certified veterinary internists to have pituitary computed tomography (CT) (Mx8000 IDT, Philips, Best, The Netherlands) performed on their cat(free of charge). During the last two years of this surveillance study, these invitations were also sent to all veterinarians taking care of cats of which the IGF-1 concentration proved between 500 and 1000 ng/ml. All CT scans were performed with the cat in sternal recumbency under deep sedation (intravenous midazolam and ketamine). Images were acquired before and immediately after administration of an intravenous bolus 2 mL/kg of iohexol (Omnipaque 300mgI/mL GE Healthcare, Norway). In CT images, the dorsal and lateral aspects of the pituitary were considered to be the interface between contrast-enhanced tissue occupying the pituitary fossa and the midbrain in images with a soft tissue window (width 200, level 80). The ventral aspect of the pituitary was considered to be in contact with the dorsal aspect of the basisphenoid bone forming the pituitary fossa, which was identified in images with a bone window (width 2500, level 500). Diagnosis of pituitary enlargement was based on observing a dorsoventral dimension of the pituitary >4.0mm and/or transverse dimension >6.0mm [15]. If the CT proved unremarkable, pituitary magnetic resonance imaging (MRI) (1.5T Intera Pulsar System, Philips Medical Systems, Reigate, UK) was subsequently offered to the owners of the cat. All MR studies were done with cats in dorsal recumbency under general anesthesia (propofol induction and isoflurane maintenance). Spin-echo T1-weighted images were acquired before and immediately after administration of an intravenous bolus of 0.1mmol/kg gadoterate meglumine (Dotarem, Guerbet, Milton Keynes, UK). CT and MR images were evaluated by a board-certified veterinary radiologist. Necropsy permission was requested at time of death and performed by a board-certified veterinary pathologist if granted. Based on current consensus in veterinary endocrinology, diabetic cats were classified as confirmed HS-induced DM on the basis of recording significant elevation of serum basal total IGF-1 concentration (>1000 ng/ml) and an enlargement of the pituitary on pituitary imaging (CT or MRI) or a structural pituitary abnormality on necropsy [16]. Clinical signs shown by confirmed acromegalic cats were compared with those shown by 20 randomly chosen cats presented with newly diagnosed primary diabetes mellitus (HS deemed unlikely based on a normal serum IGF-1 concentration) at the same Veterinary Endocrinology Clinic. This study was approved by the Royal Veterinary College’s Ethical Committee and all procedures were performed as part of the routine veterinary care of the individual animal under the Veterinary Surgeons Act applicable in the United Kingdom. The data reported in this study include the data reported in the initial pilot screening study (184 diabetic cats of which 59 with an IGF-1> 1000 ng/ml) [12] given that the current study represents an extension of that pilot study (using the same methodology) and in order to preserve the integrity of the data-set.

### Pituitary enlargement prevalence in middle-aged and older cats

In order to estimate the prevalence of pituitary enlargements unlikely related to hypersomatotropism, a board-certified veterinary radiologist (CL) analysed the pituitary size in 62 consecutive CT studies of middle-aged and older cats that presented to our veterinary hospital for imaging of the head, though explicitly not the brain (e.g. cats presenting for chronic rhinitis, otitis, jaw trauma). As per above, diagnosis of pituitary enlargement was based on observing a dorsoventral dimension of the pituitary >4.0mm and/or transverse dimension >6.0mm [15].

### Assays

All blood samples were collected via venipuncture into serum gel tubes after a minimum of 6 hours fasting and prior to exogenous insulin administration. Samples arrived at the laboratory within 24 hours of collection. Fructosamine was measured via colorimetric assay, based on the ability of ketoamines to reduce nitrotetrazolium blue to formazans in an alkaline medium and a reference interval was previously established (reference interval, 205–322 μmol/L). Basal total serum IGF-1 was measured using a radioimmunoassay system (RIA) which employs a human anti-IGF-1 antibody and is currently most commonly used for the diagnosis of HS in the cat (positive predictive value of 95% at a cut-off value of 1000 ng/mL) [12,17]. The fructosamine and IGF-1 assays were not changed during the entire evaluation period and consistency of both assays proven by ongoing intra- and inter-assay coefficient of variation (CV) surveillance (fructosamine: inter-assay CV 3.22% for a standard sample of 274 μmol/l; 3.47% for a standard sample of 557 μmol/l; intra-assay CV 1.27% for a cat sample of 204 μmol/l run 25 times; 0.96% for a cat sample of 565 μmol/l run 18 times; IGF-1: inter-assay CV 4.6% for a cat sample of 519 ng/ml; 9.3% for a standard sample of 216 ng/ml; 12.1% for a standard sample of 62 ng/ml; intra-assay CV 7.9% for a cat sample of 172 ng/ml run 18 times). Cross-reactivity of the IGF-1 assay with exogenous insulin was tested by spiking cat serum samples with a range of concentrations of the three exogenous insulin types used in the studied population (insulin lente, protamine zinc insulin and insulin glargine); cross-reactivity was not apparent.

### Statistical evaluation

Prevalence data are presented with 95% confidence intervals (CI) where appropriate. Depending on the presence of normal distribution, continuous parameters (e.g. fructosamine concentrations, body weight, insulin requirements, age) were compared by Student’s t-test or Mann-Whitney U-test respectively. The proportions of categorical data (affected breeds, gender: male/female entire, male/female neutered, clinical signs) were compared using a Chi-square (comparison of the large number of cats with IGF-1 > and < 1000 ng/ml in the overall screened population) or Fischer’s Exact test (comparison of the smaller number of confirmed acromegalic cats with regular diabetic cats). Correlations between IGF-1 and body weight were assessed by calculating a Spearman’s rho; correlation between duration of exogenous insulin administration (days) and IGF-1 by calculating Pearson’s coefficient of correlation Calculations were performed using commercial software (Statistical Package for the Social Sciences, SPSS, version 16, IBM, Portsmouth, UK and Excel, Microsoft, Redmond, USA). The significance level used in the current studies was P <0.05, unless multiple testing took place, in which case additional post hoc Bonferroni correction was applied.

## Results

### Proportion of suspected hypersomatotropism-induced DM in the diabetic cat population

Over the 9-year-period 1221 diabetic cat samples were received by the laboratory for the purpose of fructosamine evaluation; these include the 184 cat samples initially evaluated in the pilot study [12]. Three hundred and nineteen samples (26.1%) demonstrated serum total IGF-1 concentrations higher than 1000 ng/ml (95% CI: 23.6–28.6%); the remaining samples with an IGF-1 < 1000 ng/ml had a mean+/-SD IGF-1 concentration of 468+/-221 ng/ml. A comparison of characteristics between diabetic cats with high and low IGF-1 concentration (cut-off 1000 ng/ml) is shown in Table 1, depicting age, breed, gender, body weight, fructosamine concentration, exogenous insulin dose and percentage of submitting clinicians suspecting HS on the basis of clinical picture at time of sample submission. Half (50%) of the clinicians examining the cats with IGF-1< 1000 ng/ml did not suspect presence of HS (answering ‘no’), whereas a quarter (24%) of clinicians examining the cats with IGF-1>1000ng/ml strongly suspected presence of HS; the remainder was non-committal (answering ‘maybe’). The mean+/-SD duration of exogenous insulin administration in both groups was not significantly different (IGF-1<1000 ng/ml: 146+/-215 days; >1000: 133+/-200 days; P = 0.49; t-test). Additionally, there was only a very weak significant correlation between serum IGF-1 and duration of insulin administration in the <1000 ng/ml group (Pearson’s correlation coefficient: 0.0859, P = 0.0099); and no significant correlation in the >1000 ng/ml group (Pearson’s correlation coefficient: 0.000152; P = 1.0).

**Table 1:** Table comparing basic characteristics of diabetic cats with excessively high (>1000ng/ml) and low (<1000 ng/ml) serum total insulin-like growth factor-1 (IGF-1) concentration.

	Number	Age (years, mean+/-SD	Breed	Gender	Body weight (kg)	Fructosamine (μmol/l, mean+/-SD)	Insulin dose (iu/day, mean+/-SD)	Clinicians suspecting HS (%)
**IGF-1<1000 ng/ml**	902 (73.9%)	11.8+/-3.2	747 DSH^a^ (83%), 82 DLH^b^ (9%), 22 Burmese (2%), 7 Abyssinian (0.8%), 5 Maine Coon (0.6%), 5 Siamese (0.6%), 3 Persian (0.3%), 2 Bengal (0.2%), 2 British Blue (0.2%), 2 Russian Blue (0.2%), 10 other breeds (1%)	588 MN^d^ (65%)14 ME^e^(1.6%)290 FN^f^ (32%)7 FE^g^(0.8%)	5.2+/-1.4	476+/-152	5.8+/-5	No: 450 (50%)Maybe: 179 (20%)Yes: 10 (1%)
**IGF-1>1000 ng/ml**	319(26.1%)	11.3+/-2.7	271 DSH (85%), 22 DLH (7%), 5 Burmese (2%), 5 Maine Coon (1.6%), 2 BSH^c^ (0.6%)	226 MN^d^ (71%)8 ME^e^ (2.5%)82 FN^f^ (26%)2 FE^g^ (0.6%)	5.4+/-1.3	560+/-142	13.8+/-10	No: 47 (15%)Maybe: 127 (40%)Yes: 78 (24%)

^a^Domestic Short Hair Cat

^b^Domestic Long Hair Cat

^c^British Short Hair Cat

^d^male neutered

^e^male entire

^f^female neutered

^g^female entire.

Diabetic cats with IGF-1 greater than 1000 ng/ml demonstrated a higher body weight (Mann Whitney test, p = 0.015), lower age (Mann Whitney test, p = 0.03), higher fructosamine concentration (Mann Whitney test, p<0.001), as well as higher administered insulin dose per day (Mann Whitney test, p<0.001), though only the latter two features proved significant after post hoc Bonferroni correction. Although proportionately three times as many Maine Coon cats were represented in the IGF-1>1000 ng/ml group, cat breed distribution did not prove significantly different between the two groups (Chi-square, p = 0.23), nor did gender distribution (Chi-square, p = 0.13). In diabetic cats with IGF-1<1000 ng/ml, a small but significant correlation existed between body weight and IGF-1 concentration (Spearman’s rho 0.34, p<0.001), which did not exist in those with IGF-1>1000 ng/ml (Spearman’s rho -0.07, p = 0.294).

### Confirmation of hypersomatotropism-induced DM

**Fig 1:**
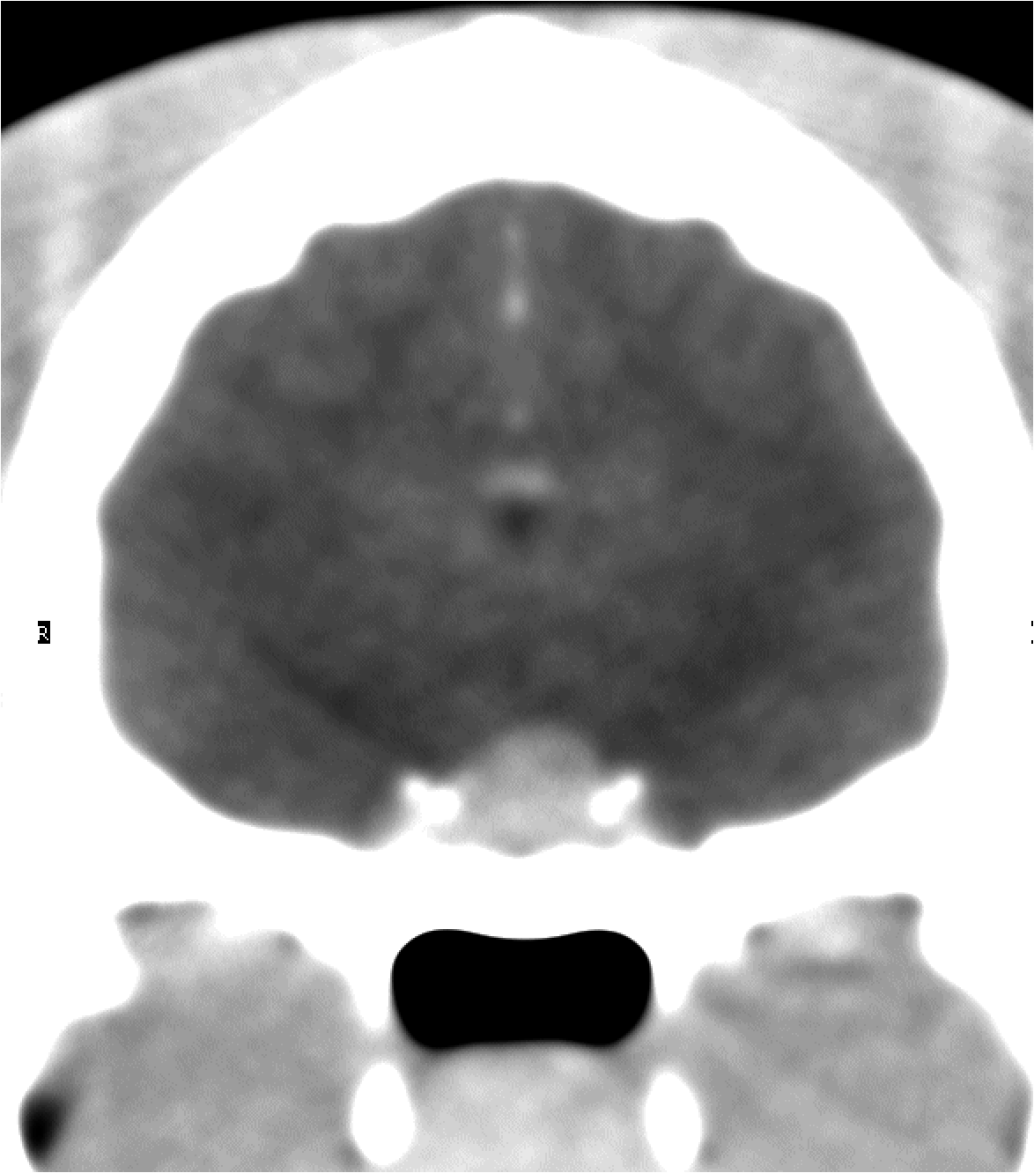
Protrusion of the pituitary beyond the dorsal rim of the sella turcica. This figure shows an example of a transverse, post-contrast CT image in a cat with HS. In this instance the dorsoventral dimension (height) of the enlarged pituitary was 6mm.

**Fig 2:**
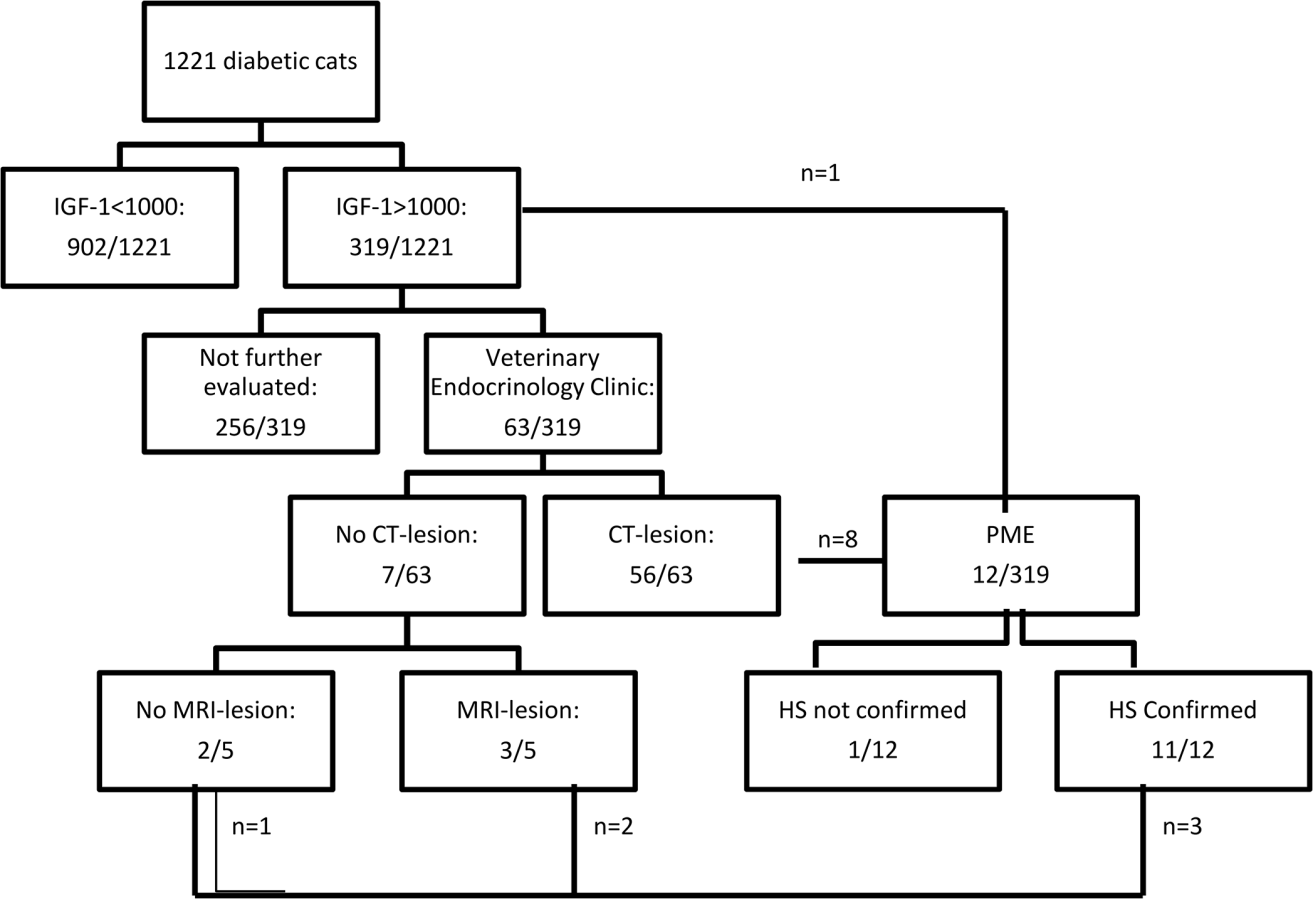
Overview of the screening results. In total 1221 cats with diabetes mellitus were screened for HS; the results are summarised in this figure. IGF-1: insulin-like growth factor-1; CT: computed tomography; MRI: magnetic resonance imaging; HS: hypersomatotropism; n: number of animals; PME: post-mortem evaluation.

Sixty-three of 319 diabetic cats (20%) from the suspected HS-induced DM category were available for a visit to the Veterinary Endocrinology Clinic and subsequent intracranial imaging. Fifty-six of these 63 cats (89%) had a demonstrable pituitary lesion on CT imaging (Fig 1). Out of the seven cats that did not show such lesion, five subsequently underwent intra-cranial MR imaging, which demonstrated a lesion in the pituitary in three cats, additionally confirming presence of HS in these cases. Of the two cats with no lesion on CT or MR imaging, one underwent necropsy which demonstrated acidophilic hyperplasia (this cat formed part of the pilot study [12]). Altogether 60 of the 63 assessed cases were confirmed to have HS. The positive predictive value of the employed IGF-1 RIA for the diagnosis of HS in the cat at a cut-off of 1000 ng/ml was therefore calculated to be 95% (95% CI: 90–100%). A systematic overview of these findings is depicted in Fig 2. This calculated positive predictive value for serum IGF-1 concentration > 1000 ng/ml therefore suggests the overall proportion of HS-induced DM cats in the greater studied population of 1221 diabetic cats to be 24.8% (95% CI: 21.2–28.6%).

Additionally, 11 of 372 diabetic cats (3.0%) with an IGF-1 > 500 ng/ml, though <1000 ng/ml were recruited to have a CT-study of their pituitary performed (median IGF-1: 795 ng/ml, range: 526–986 ng/ml). One of these 11 cats (9.1%) had a demonstrable pituitary enlargement on CT imaging. This cat had the highest IGF-1 concentration, close to the 1000 ng/ml cut-off (986 ng/ml). On the basis of these data, the negative predictive value of the employed <1000 ng/ml cut-off value for the diagnosis of HS in the diabetic cat is 91% (95% CI: 74–100%).

### Pituitary enlargement prevalence in middle-aged and older cats

Mean pituitary height in 62 consecutive CT studies of cats presented for imaging of the head, though explicitly not the brain, was 3.1+/-0.4 mm (SD). Only one cat displayed an increased pituitary size, with a pituitary height of 4.2 mm. This suggests the prevalence of incidental pituitary enlargement in older cats to be 1.6% (95% CI: 0–4.7%).

### Clinical presentation of confirmed hypersomatotropism-induced DM cases

**Fig 3:**
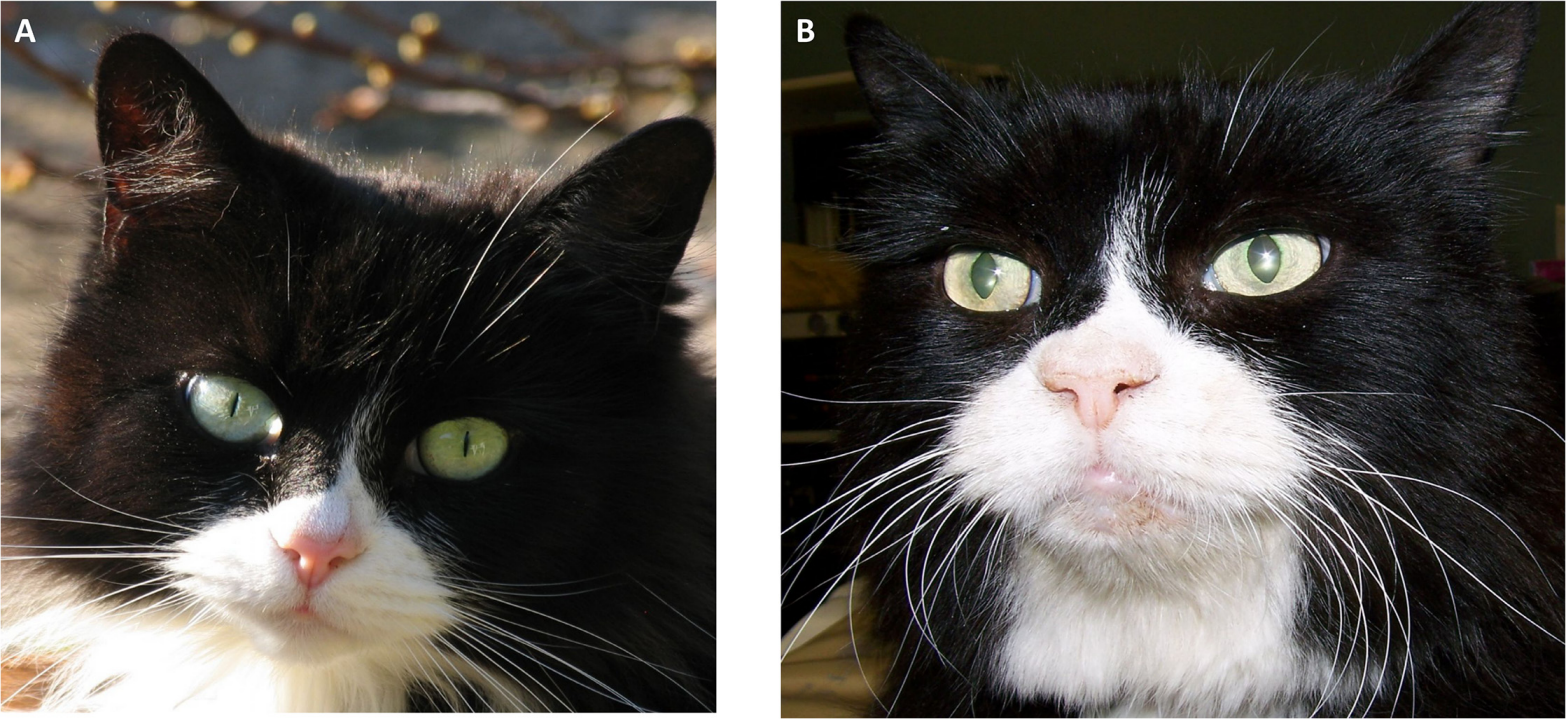
Example of a cat before and after onset of HS-induced changes. Photo a was taken in 2009 and photo b after onset of HS-induced changes in 2012. HS-induced broad facial features are evident in the later picture.

**Fig 4:**
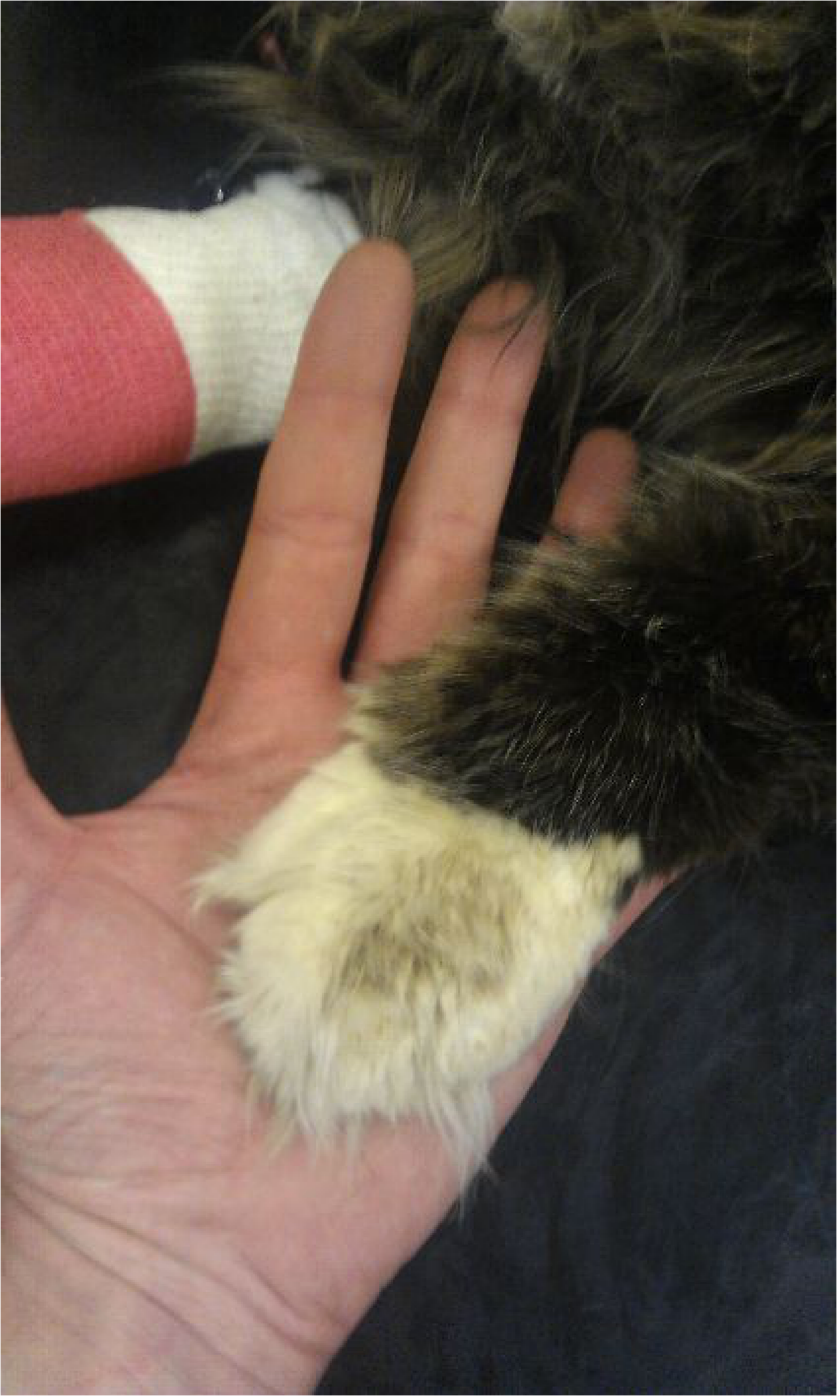
Paws of an overtly acromegalic cat. This figure shows a cat with confirmed acromegaly demonstrating so-called clubbed paws; enlargement of the paws due to acromegaly.

**Fig 5:**
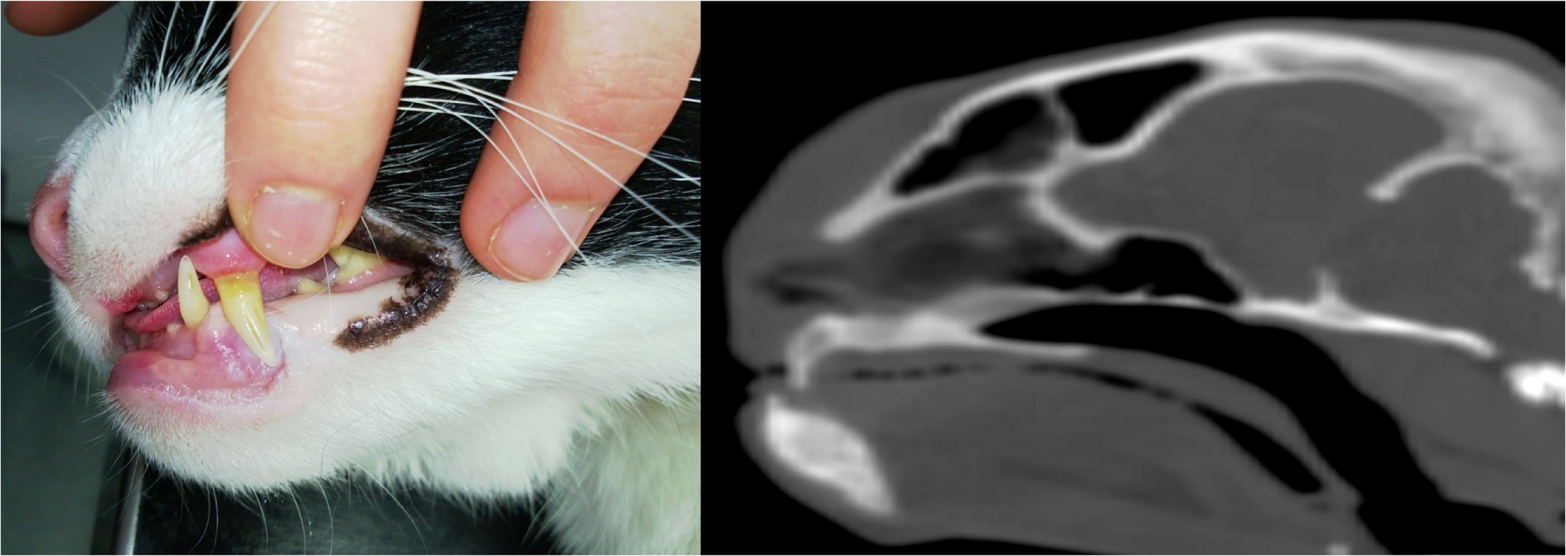
Example of prognathia inferior. This figure shows a cat with confirmed acromegaly demonstrating prognathia inferior (a), including a sagittal CT view of the cat’s head illustrating this conformational change further (b).

**Fig 6:**
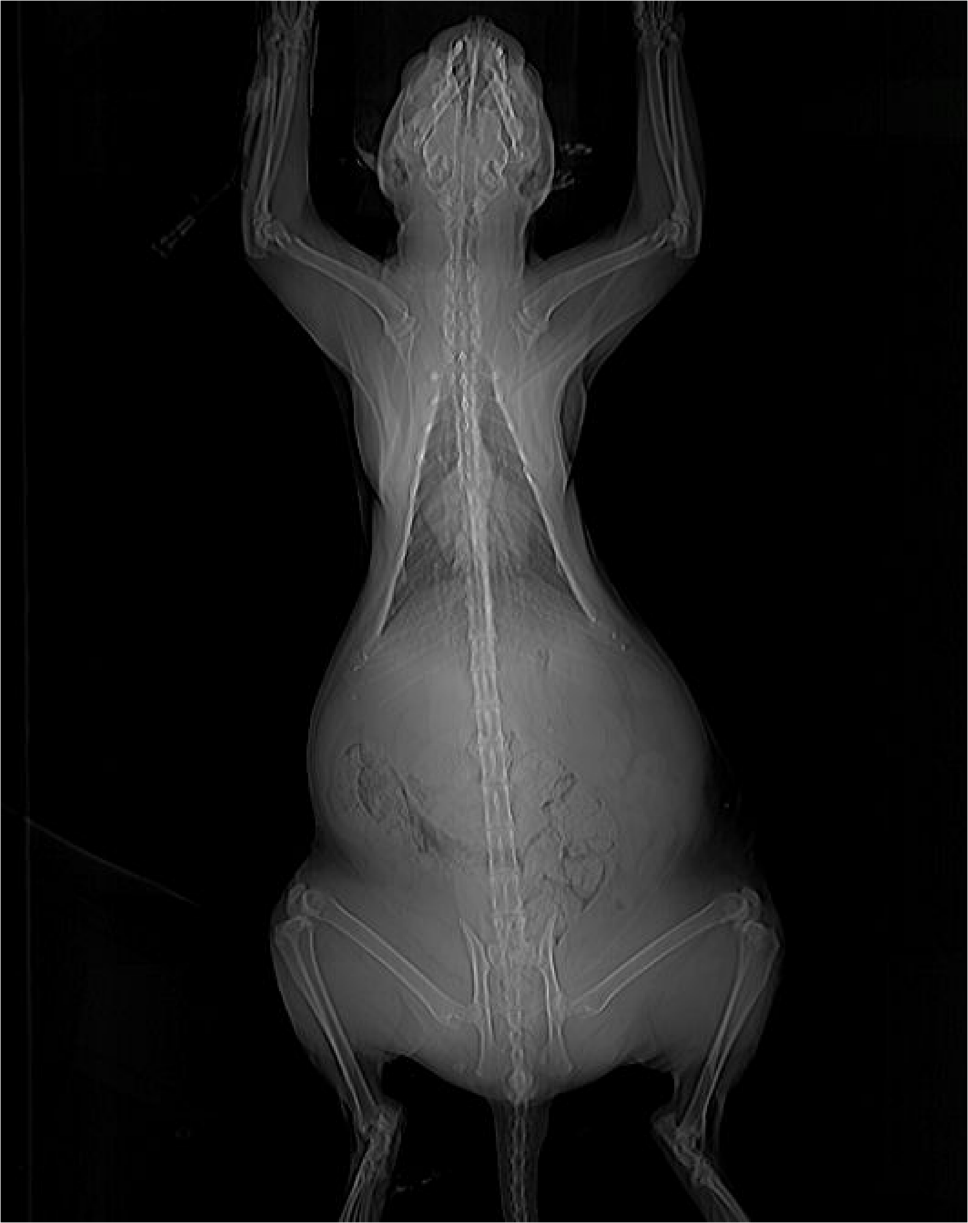
Radiograph of the chest and abdomen of an overtly acromegalic cat. Cranial abdominal organomegaly was demonstrated by this radiographic view of the chest and abdomen of a diabetic cat subsequently diagnosed with HS.

The clinical details (clinical signs reported by cat owner and Veterinary Endocrinology Clinic clinicians, including physical examination abnormalities suggestive of HS) of the 60 cats with confirmed HS are summarised in Table 2. Most cats demonstrated signs compatible with diabetes mellitus, including polyuria, polydipsia and polyphagia. Phenotypical abnormalities historically associated with feline HS and which form part of the clinical syndrome of acromegaly, including prognathia inferior (protrusion of the mandible), weight gain, abdominal organomegaly, broadening of paws (so-called clubbed paws) and face were present in only a minority of cases. Examples of some of the obvious phenotypical changes encountered in a minority of cats are shown in Figs 3–6. Compared to a diabetic control group without HS (based on normal serum IGF-1), the following clinical features were statistically overrepresented in the HS group: extreme polyphagia (p = 0.031), respiratory stridor/ snoring (p = 0.025) and broad facial features (p<0.001), though only the latter feature proved significant after post hoc Bonferroni correction (Table 2).

**Table 2:** Table depicting the clinical signs displayed by the diabetic cats in the group with confirmed HS, compared to a group of cats newly diagnosed with primary diabetes mellitus (non-acromegalic).

n = 60	Number of diabetic cats with confirmed hypersomatotropism in which this was present (percentage) n = 60	Number of diabetic cats without HS in which this was present (percentage) n = 20	Fischer’s exact test result comparing the frequency clinical sign (p = value)
**Polyuria**	52 (87%)	15 (75%)	0.29
**Polydipsia**	52 (87%)	17 (85%)	1.0
**Polyphagia**	45 (75%)Of which extreme: 12 (20%)	11 (55%) Of which extreme: 0 (0%)	0.10**0.031**
**Weight loss**	25 (42%)	12 (60%)	0.20
**Weight gain**	10 (17%)	0 (0%)	0.059
**Respiratory stridor / ‘snoring’**	23 (38%)	2 (10%)	**0.025**
**CNS signs (excluding lethargy)**	1 (1.7%)	0 (0%)	1.0
**Lethargy**	15 (25%)	7 (35%)	0.40
**Stiffness / mobility problems**	6 (10%)	2 (10%)	1.0
**Abdominal organomegaly (renomegaly and/or hepatomegaly**	24 (40%)	5 (25%)	0.29
**Prognathia inferior (Fig 4)**	11 (18%)	2 (10%)	0.50
**Clubbed paw appearance (Fig 5)**	8 (13%)	0 (0%)	0.19
**Broad facial features (Fig 6)**	22 (37%)	0 (0%)	**<0.001**
**Heart murmur**	11 (18%)	4 (20%)	1.0
**Plantegrade stance**	2 (3%)	2 (10%)	0.26

In bold: significant p-value, not corrected for multiple comparisons; in bold and underlined: significant p-value after Bonferroni post-hoc correction for multiple comparisons.

### Necropsy

**Fig 7:**
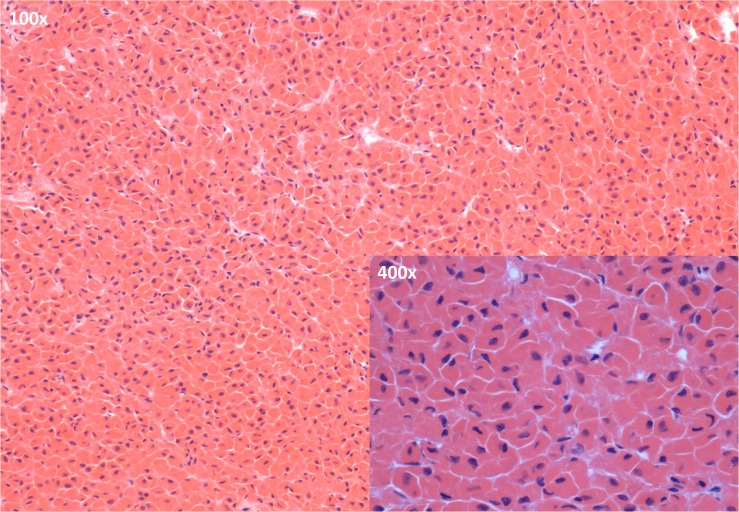
Microscopic image of the anterior pituitary of a diabetic cat with confirmed HS. A clear predominance of acidophils is apparent in the sections of the pars distalis of the anterior pituitary (H&E staining; main image: objective 100x; inset: objective: 400x)

**Fig 8:**
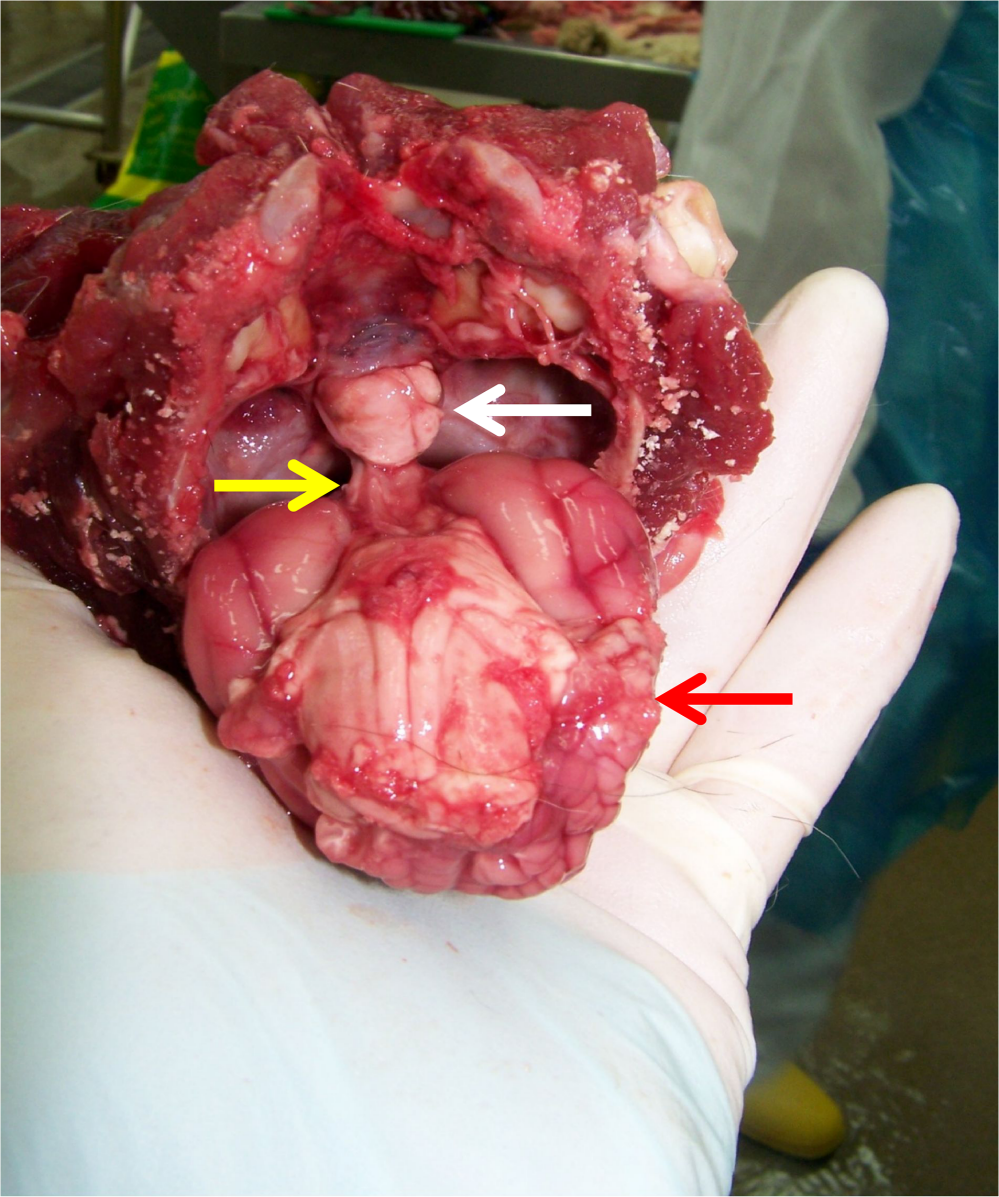
Macroscopic necropsy picture of the brain of a diabetic cat with confirmed HS. This picture shows the ventral aspect of the brain of a cat with HS as it was removed from the cranial cavity and a clearly enlarged pituitary falling out of the sella turcica (cranium is held upside down; white arrow: pituitary gland; yellow arrow: pituitary stalk; red arrow: cerebrum). Histopathology confirmed the presence of an acidophilic macroadenoma.

Macroscopic and microscopic necropsy of the pituitary was performed in 12 of 319 cases of suspect and/or confirmed HS-induced DM (3.8%), 11 of which underwent the above ante-mortem intracranial imaging (Figs 7 and 8). HS could be convincingly confirmed in all 11 of these latter cases on the basis of visualisation of acidophil hyperplasia (n = 4 of which one was previously described [12]), adenoma (n = 6 of which 2 were previously described [12], Fig 8) or adenocarcinoma (n = 1). The pituitary appeared normal on histopathology in one case with IGF-1 > 1000 ng/ml without prior intra-cranial imaging (this case was previously described as part of the initial pilot study [12].

## Discussion

It has been common practice to automatically suspect a cat with DM to be suffering from a form of diabetes akin to human type 2 DM. The current study suggests that this is an oversimplification. Approximately one in four of assessed diabetic cats were found to be suffering from HS-induced DM, which has a distinctly different aetiopathogenesis. HS therefore seems more common in the cat with DM, compared to humans, though its prevalence among humans with DM and glucose intolerance is similarly thought to be underestimated [18,19]. This is in line with the results of a small pilot study the authors previously performed using the same methodology [12]. In light of this high prevalence and the demonstrated lack of ease of phenotypic recognition of HS in the diabetic cat, it is recommended that any future study using spontaneous feline DM as a comparative model for human type 2 DM should consider screening for the disease. The same advice seems appropriate for veterinarians trying to manage DM in cats, given the significant clinical consequences of its presence. Indeed, should the HS be diagnosed and treated, most cats will enter a state of diabetic remission [16]. If the HS remains undiagnosed, these diabetic cats tend to be difficult to regulate glycaemically. Given the financial limitations in veterinary medicine, lack of prompt glycaemic control often results in euthanasia. If kept alive, acromegalic cats will, in the long-term, suffer from other growth hormone-induced negative sequelae, as well as pituitary tumour induced central nervous signs [16].

In line with Melmed’s [18] and Rosario’s [19] findings in man, the current study also shows that the measurement of total serum IGF-1 can be useful for the purpose of screening diabetic cats for acromegaly, given its positive predictive value of 95% at a cut-off of 1000 ng/ml. The negative predictive value was estimated to be 91% at this same cut-off. Admittedly, the one case that proved to have a pituitary enlargement despite an IGF-1<1000 ng/ml had an IGF-1 very close to this cut-off (986 ng/ml). Pituitary enlargement appeared to be rare in the general cat population (1.6%) on the basis of CT-evaluation of cats presented for other reasons than pituitary assessment. This further suggests that the calculated positive predictive value of an IGF-1 > 1000 ng/ml is accurate and unlikely an overestimation due to misclassification of incidental or non-functional pituitary enlargements.

It is recognised that circulating IGF-1 concentration can be influenced by various parameters. For example, portal insulin is required for hepatic IGF-1 production. Since, in parallel with people suffering from DM, endogenous insulin concentrations can be low in feline DM, a low IGF-1 reading could be obtained when sampling takes place prior to initiation of exogenous insulin treatment despite the presence of excess growth hormone due to acromegaly [16, 20]. For this reason, the current estimation of the prevalence of HS using IGF-1 as a screening test could represent an underestimation. Duration of exogenous insulin administration has been shown to affect circulating total IGF-1 concentrations [21–23]. The absence of a difference in duration of exogenous insulin administration between the > and < 1000 ng/ml IGF-1 groups, suggests that this factor did not pose a confounding factor that likely could have influenced the current study’s main findings. Furthermore, the calculated Pearson correlation coefficients suggested that in the studied population, any influence of duration of insulin administration on IGF-1 concentrations to be rather small.”

Alternatively, reasons also exist why the current prevalence figure could be an overestimation. These include the fact that recruitment of diabetic cat samples was driven by the fact that veterinarians could obtain a free fructosamine evaluation as a measure of the cat’s glycaemic control; the excess serum of these samples was used to determine IGF-1. Measuring fructosamine is a common practice in both stable and unstable diabetics in veterinary medicine, though this might have been of added interest in those cases whose DM proved more difficult to control. Acromegalic diabetic cats can prove more difficult to control from a DM point of view [11] and samples of these cats could therefore have been preferentially sent in. Nevertheless, the wide range of fructosamine concentrations in both the IGF-1<1000 ng/ml and the IGF-1>1000 ng/ml group suggests that overall a population with a mix of stable and unstable diabetic control was sampled. Finally, in the world of veterinary medicine, it would have been near impossible to test the unprecedented number of diabetic cat samples assessed in the current study without having provided an incentive related to the cats’ diabetic management. Pituitary imaging proved useful in the assessed cases, although, in parallel with the situation in humans, both CT and MRI failed to detect a minority of cases due to the presence of only microscopic changes.

In people the gold standard diagnosis of acromegaly involves performing a GH suppression test through intravenous administration of glucose [18]. This suppression test has currently not been validated for use in the cat and might not prove useful, since previously no suppression was shown to occur in four healthy cats [24]; further assessment of this test is currently hampered by the absence of a commercially available feline GH assay. Human GH assays cannot be used given the great inter-species heterogeneity of this hormone. Instead, therefore, serum IGF-1 was used, which has been proven to be a relatively accurate diagnostic tool for establishing the presence of acromegaly both in humans [18,19] and the cat [16].

Interestingly, only a small proportion (24%) of clinicians submitting samples of cats which subsequently proved to be at high risk of having acromegaly (IGF-1 > 1000 ng/ml), reported they strongly suspected acromegaly to be present on the basis of the clinical picture. This is likely linked to the fact that acromegaly, like in humans [25], is a gradually progressive disease in cats. The occurrence of DM often precedes the soft tissue and bony changes classically associated with the disease in cats and diabetic signs are more acute in onset, as well as easier to spot for cat owners. This implies that an acromegalic diabetic cat can easily be mistaken for a non-acromegalic diabetic cat when only judging the phenotype. This is further demonstrated in the current study, with only 40% of confirmed acromegalic cats displaying abdominal organomegaly, 18% showing prognathia inferior, 13% “clubbed paws” and 37% broad facial features (of which only the latter proved statistically significantly increased in prevalence compared to a diabetic control group). The occurrence of respiratory stridor / snoring in cats with HS likely reflects reduced diameter of the nasopharynx, which occurs apparently as a result of thickening of soft tissues of the head [26].

The low prevalence of phenotypical changes suggestive of GH excess is likely also due to the study design. The proactive screening approach adopted in the current study with as only requirement presence of DM, likely resulted in acromegalic cases being diagnosed early on in the disease process with little time having passed for GH and IGF-1 to exert their anabolic and catabolic effects. In cats, additional difficulties recognising signs of acromegaly may occur because of facial hair and the tendency for owners to examine their pet’s face infrequently. A recent study included examples of 3-dimensional surface-rendered CT images, which display the skin surface without hair, which enables a truer view of the surface contours. Even so, distinguishing affected and unaffected cats on the basis of their facial features is difficult [26]. A lack of awareness among both veterinary and medical professionals could further enhance any underdiagnosis.

The current study also emphasises that the acromegalic cat could prove useful as a spontaneous model for the human form of the disease, especially given the fact that several factors are shared by both species. These include: a shared environment, a pituitary acidophilic adenoma as the most common cause of the growth hormone hypersecretion and, ultimately, occurrence of similar physical changes in chronic cases, including prognathia inferior, broad facial features, abdominal organomegaly, cardiomegaly and arthropathy. The prevalence of acromegaly in the human population is relatively low, with estimates stated to be in the region of 40–128 cases/1,000,000 population [27, 28]. As a consequence, basic and clinical acromegaly research suffers somehow from relatively slow recruitment of patients as well as biological samples. On the other hand, with a prevalence of 26.1% among diabetic cats and an estimated prevalence of diabetes of 1 in 200 cats in the UK [1] up to one and a half in 200 in the United States [3], the overall estimated prevalence of acromegaly in the domestic cat could be significantly higher than in the human population. The potential of feline acromegaly providing novel angles to comparative acromegaly research was demonstrated in a recent study looking into the relationship between environmental toxin exposure and occurrence of endocrinopathies, using the acromegalic cat as a spontaneous model for the human disease. The study found that higher levels of organohalogenated chemicals, which can be found in any household and can induce pituitary oncogenesis, were present in the plasma of acromegalic household cats [4]. Additionally, at least as interesting would be the study of those factors that are different in both species. Particularly the seemingly lower prevalence of DM in man springs to mind. The prevalence of DM among those suffering from acromegaly varies according to the study examined, though seems broadly in the range of 19–56% [18,19]. This variation is likely due to the difference in the studied populations, including ethnicity [18]. Higher GH concentrations, increasing age and longer disease duration significantly predicted the tendency of developing symptomatic diabetes mellitus in humans [29,30]. The seemingly stronger association between acromegaly and diabetes in the cat could therefore be due to the fact that acromegalic cats only get presented to a veterinary practice at a late stage in the disease process. What would argue against that, however, would be the relatively low prevalence of phenotypical signs the diabetic acromegalic cats present with. Additionally, many acromegalic cats once effectively treated for the acromegaly, will go into diabetic remission, possibly suggesting their endocrine pancreas has not yet been subjected to overly long periods of the insulin resistance associated with acromegaly, leading to beta-cell exhaustion [16]. Interestingly, currently no case report exists in the literature of a non-diabetic acromegalic cat. In light of the above, there remains margin for yet to be identified processes to be at work, explaining this higher prevalence of DM among feline acromegalics. This encountered stronger association between feline acromegaly and feline DM, compared to the human equivalents, could harbour novel information with regards to our understanding of the interaction between growth hormone, beta-cell dysfunction and insulin resistance.

It could be argued that the DM was in fact a co-morbidity of the patients who happened to also be diagnosed with acromegaly. Nevertheless, the authors deem it more likely that the DM seen in these cats was induced by acromegaly. This is substantiated by the fact that the DM often disappears if the acromegaly is effectively treated through hypophysectomy, radiotherapy or somatostatin treatment [16, 31, 32, 33]. Furthermore, the diabetes-type that the acromegalic cats suffer from seems to be more insulin-resistant than non-acromegalic diabetic cats, as illustrated by the significantly higher exogenous insulin requirements among acromegalic cats found in this study.

Interestingly, although most cases submitted for post mortem showed evidence of an acidophilic adenoma, 4/11 post mortem cases with confirmed acromegaly demonstrated acidophilic hyperplasia. The distinction between hyperplasia and micro-adenoma can be arbitrary on microscopic examination of endocrine tissues and a continuum may exist between the two processes [34]. Excess production of growth hormone–releasing hormone has been rarely described to result in somatotroph hyperplasia and acromegaly [35]. Both central hypothalamic tumours (usually gangliocytomas) and peripheral neuroendocrine tumours could secrete growth hormone–releasing hormone (GHRH), inducing somatotroph proliferation (and very rarely, the formation of an adenoma), with resultant elevations in levels of growth hormone and IGF-I [18]. Evidence of neuroendocrine tumours or a hypothalamic lesion were not detected during the thorough necropsies, despite macro- and microscopic evaluation of a wide range of tissues (including hypothalamus and pancreas). An alternative explanation could lay in the fact that microscopic classification and differentiation between adenoma and hyperplasia remains notoriously challenging. Assessment of clonality of cells, as well as evaluation of circulating GHRH concentrations might have helped, all of which are unfortunately not currently available for the domestic cat.

## Conclusions

The current study suggests that HS caused by a pituitary adenoma or hyperplasia is common in the domestic cat with DM and explains the presence of DM in approximately 1 in 4 cats. Cats with HS often display an unremarkable phenotype, indistinguishable from cats with primary (type 2) DM. The current data therefore provide a warning for researchers and veterinarians working with the spontaneously diabetic cat to consider routinely screening such cats for the presence of HS-induced DM; serum IGF-1 was confirmed to be a relatively good tool for this. The encountered high prevalence and similarities between feline and human acromegaly emphasise great opportunities exist for valuable comparative acromegaly research benefiting all species suffering from this condition, including man.
